# Exiting the pandemic together: achieving global immunity and equity

**DOI:** 10.18632/oncoscience.585

**Published:** 2023-09-01

**Authors:** Yuxin Ying, Jola Bytyci, Lennard YW Lee

**Keywords:** COVID-19, immunocompromised, vaccination, breakthrough infection, cancer

The effectiveness of the third booster vaccine was investigated in a recent paper by LWY Lee et al. titled ‘COVID-19: Third dose booster vaccine effectiveness against breakthrough coronavirus infection, hospitalisations and death in patients with cancer: A population-based study’ [1]. Overall, the study found that the third dose booster improved vaccine effectiveness across various measures. However, the benefits of the booster were not as significant for patients with cancer when compared to the general population. This is because immunocompromised (IC) individuals, including those with cancer, have a diminished response to vaccination [1]. As a result, they continue to remain at a high risk of experiencing breakthrough infections and severe cases of COVID-19. Unfortunately, vaccination alone does not provide an adequate level of protection for these groups [2]. Consequently, additional measures such as prophylactic antibodies are internally agreed to be the standard of care to address the ongoing impact of the pandemic on affected individuals [3].

This study represents the largest global evaluation of the efficacy of the third dose booster vaccine against SARS-CoV-2. The primary outcomes were to assess vaccine effectiveness against breakthrough infections, symptomatic infections, hospitalisation, and death. The study period spanned from August 12th 2020 to July 12th 2021, which coincided with the start of COVID-19 vaccination in England. The data set used for analysis included all COVID-19 PCR tests from England. This assessment was performed following the administration of both the second and third dose of the vaccine. The cancer cohort consisted of adults from the NHS cancer registry between January 1st 2019 and April 30th 2021 whilst the population control group comprised tests from adults not included in the national cancer dataset [1].

The results revealed that third dose boosters increased vaccine effectiveness against all primary outcome measures in both cohorts. However, the benefits of the third dose boosters were found to be less substantial in patients with cancer compared to the general population, with variations in response noted between individuals with different cancer subtypes. For example, individuals with solid organ malignancies experienced higher vaccine effectiveness against breakthrough and symptomatic infections compared to those with haematological malignancies. Interestingly, patients with lymphoma exhibited low levels of vaccine effectiveness against breakthrough and symptomatic infections, which did not improve following the administration of a third vaccine dose. However, the third dose booster did provide higher levels of protection against more severe outcomes, such as hospitalisation and death [1].

LWY Lee et al. also identified certain factors associated with lower vaccine effectiveness in patients with cancer including a recent cancer diagnosis or receipt of systemic anticancer therapy (SACT) or radiotherapy within the past 12 months. To account for potential confounding variables, a multivariable logistic regression model was employed adjusting for age, sex, levels of deprivation, ethnicity, primary and booster dose manufacturer. The analysis indicated that the cancer cohort was not at an increased risk of breakthrough infections. However, following a positive test, individuals in the cancer cohort did face an elevated risk of hospitalization and death compared to the control group [1].

This comprehensive evaluation sheds light on the varying effectiveness of third dose boosters in individuals with cancer and emphasizes the importance of considering additional factors when assessing vaccine effectiveness in this population.

Several studies have shown that IC individuals, including cancer patients continue to be affected by the pandemic, supporting the main conclusions made by LWY Lee et al. Numerous other studies have demonstrated that breakthrough infections against SARS-CoV-2 are higher in patients with cancer compared to non-cancer patients [4–6]. The risk of severe COVID-19 outcomes (hospitalisation and death) following breakthrough infection has also been reported to be higher in cancer patients versus non-cancer patients, supporting the findings of Lee et al. [5]. Collectively, these findings emphasize that despite vaccination, cancer patients remain at risk of COVID-19 breakthrough infections and severe outcomes.

There are data showing that IC individuals remain at risk of breakthrough infections and severe COVID-19 outcomes as a result of a weakened immune response to vaccination compared to the general population [2]. Consequently, IC patients continued to maintain shielding behaviours to reduce their risks well into the post-vaccination era. The final report on Coronavirus and clinically extremely vulnerable (CEV) people in England from the UK census stated that in April 2022, 13% of individuals previously considered CEV to COVID-19 continued to follow shielding advice, and 69% took extra precautions [7]. Moreover, 46% of CEV individuals reported feeling worried about the impact of COVID-19 on their lives, compared to 34% of the general adult population in England [7].

While vaccination have been successful for the general population, it is crucial not to overlook the needs of immunocompromised individuals. It is necessary to implement appropriate measures to protect and support their reintegration into society. Failure to do this would mean that the liberties of the population would have occurred at the expense of vulnerable groups. Additional measures would include considering additional booster vaccinations, with further research into their efficacy. Furthermore, there is a need for initiatives to advance vaccine adjuncts, second generation vaccines or prophylactic antibodies to provide the necessary support and protection for immunocompromised individuals in the face of the ongoing pandemic.
